# Sex dimorphic cortical brain volumes associated with antisocial behavior in young adults

**DOI:** 10.1093/psyrad/kkad031

**Published:** 2023-12-19

**Authors:** Ke Ding, Miao Xu, Taicheng Huang, Yiying Song, Feng Kong, Zonglei Zhen

**Affiliations:** School of Psychology, Shenzhen University, Shenzhen 518060, China; Beijing Key Laboratory of Applied Experimental Psychology, Faculty of Psychology, Beijing Normal University, Beijing, China; State Key Laboratory of Cognitive Neuroscience and Learning & IDG/McGovern Institute for Brain Research, Beijing Normal University, Beijing 100875, China; Department of Psychology and Tsinghua Laboratory of Brain and Intelligence, Tsinghua University, Beijing 100084, China; Beijing Key Laboratory of Applied Experimental Psychology, Faculty of Psychology, Beijing Normal University, Beijing, China; State Key Laboratory of Cognitive Neuroscience and Learning & IDG/McGovern Institute for Brain Research, Beijing Normal University, Beijing 100875, China; School of Psychology, Shaanxi Normal University, Xi'an 710119, China; Beijing Key Laboratory of Applied Experimental Psychology, Faculty of Psychology, Beijing Normal University, Beijing, China; State Key Laboratory of Cognitive Neuroscience and Learning & IDG/McGovern Institute for Brain Research, Beijing Normal University, Beijing 100875, China

**Keywords:** antisocial behavior, sex differences, magnetic resonance imaging, voxel-based morphometry

## Abstract

**Background:**

Although sex differences in antisocial behavior are well-documented, the extent to which neuroanatomical differences are related to sex differences in antisocial behavior is unclear. The inconsistent results from different clinical populations exhibiting antisocial behaviors are mainly due to the heterogeneity in etiologies, comorbidity inequality, and small sample size, especially in females.

**Objective:**

The study aimed to find sexual dimorphic brain regions associated with individual differences in antisocial behavior while avoiding the issues of heterogeneity and sample size.

**Methods:**

We collected structural neuroimaging data from 281 college students (131 males, 150 females) and analyzed the data using voxel-based morphometry.

**Results:**

The gray matter volume in three brain regions correlates with self-reported antisocial behavior in males and females differently: the posterior superior temporal sulcus, middle temporal gyrus, and precuneus. The findings have controlled for the total cortical gray matter volume, age, IQ, and socioeconomic status. Additionally, we found a common neural substrate of antisocial behavior in both males and females, extending from the anterior temporal lobe to the insula.

**Conclusion:**

This is the first neuroanatomical evidence from a large non-clinical sample of young adults. The study suggests that differences in males and females in reading social cues, understanding intentions and emotions, and responding to conflicts may contribute to the modulation of brain morphometry concerning antisocial behavior.

## Introduction

Antisocial behavior is a spectrum of behaviors ranging from less severe, non-aggressive rule-breaking behaviors, such as lying, to the very critical, violent, and aggressive behaviors as reported in criminal records. These behaviors have added burdens to the educational, medical, and judicial systems, as well as bringing afflictions to the victims, families, and society. Therefore, antisocial behavior has always been under the spotlight of public concerns; and it has attracted research interests from many diverse disciplines such as pedagogy, psychology, sociology, and psychiatry (Bennett *et al*., 2005; Blair, 2001; Dabbs & Morris, 1990; Moffitt *et al*., 2001; Patterson *et al*., 1989; Raine *et al*., 2011).

Of all the studies from those diverse disciplines, one consensus was reached: antisocial behavior is more prevalent in males than in females throughout lifespan; and such male preponderance was found consistently in terms of behavioral frequency, quantity, and severity (Bennett *et al*., 2005; Blair, 2001; Dabbs & Morris, 1990; Moffitt *et al*., 2001; Patterson *et al*., 1989). In particular, males have an earlier onset of antisocial behavior and commit more antisocial behavior in adolescence; moreover, life-course-persistent antisocial behavior is 10 to 14 times more prevalent in males than in females (Moffitt *et al*., 2001; Yildirim & Derksen, 2012).

Although the observed behavioral differences are well-documented, the etiology of the sex differences on antisocial behavior is still unclear. For example, most twin-family studies have reported no sex differences on the genetic and environmental contributions to antisocial behavior (Burt *et al*., 2019). Specifically, for the neuroanatomical substrates, only five studies so far have reported the structural differences between males and females relevant to antisocial behavior (Fairchild *et al*., 2013; Ibrahim *et al*., 2021; Michalska *et al*., 2015; Raine *et al*., 2011; Smaragdi *et al*., 2017). One study was on adults with antisocial personality disorder, and the rest were on children and adolescents with disruptive behavior disorders, which included conduct disorder and oppositional defiance disorder. Studies have reported sex-by-diagnosis interactions within various brain regions. Notably, in the frontal cortex, interactions have been observed in the superior frontal gyrus (Smaragdi *et al*., 2017), middle frontal gyrus, orbitofrontal cortex (Raine *et al*., 2011), and ventromedial prefrontal cortex (Ibrahim *et al*., 2021). Similarly, in the temporal and parietal cortex, the superior temporal sulcus (Michalska *et al*., 2015) and supramarginal gyrus (Ibrahim *et al*., 2021) exhibited such interactions. Furthermore, additional regions such as the anterior insula (Fairchild *et al*., 2013), amygdala (Ibrahim *et al*., 2021), and parahippocampal cortex (Smaragdi *et al*., 2017) have also been reported to display the interactions. However, there is little consensus on the brain structures specific to the sex differences relevant to antisocial behavior, and the divergence on a same finding is hard to explain. This could be due to the differences in disease-specific etiologies, comorbidities, medication, age, small sample size of the female patients, and other control factors such as IQ and socioeconomic status (SES).

Therefore, in the current study, we aim to examine the associations between sex differences in brain structure in males and females and antisocial behavior severity in a college sample. In particular, we are interested in finding brain regions in which gray matter volume covaries with the individual differences in self-reported antisocial behavior in males and females differently. To further validate the findings, we tested whether the effects were still there after regressing out other variations in age, IQ, SES, and total cerebral gray matter volume. Besides, we also checked the common neural correlates of antisocial behavior for males and females.

## Methods

### Participants

A total of 281 undergraduates from Beijing Normal University participated in the study, including 131 males and 150 females. All participants had no diagnosed mental disorder history, such as conduct disorder and antisocial personality disorder, neurological diseases (epilepsy, brain injury, neurodegenerative diseases, and cerebrovascular diseases), or any intellectual disability before taking the tests. All behavioral and magnetic resonance imaging (MRI) experiments were approved by the Ethics Committee of Beijing Normal University, and informed consents were obtained prior to the experiments. One participant was excluded from data analysis because of incomplete data on the antisocial questionnaire, and six other participants were removed because they did not participate in the following IQ test or the SES questionnaire. One participant was excluded because his score on antisocial behavior fell out by three standard deviations; similarly, another participant was excluded because the IQ score fell out by three standard deviations. In addition, six other participants had excessive head movement during the scan and therefore were not included in the analyses. Taken together, we reported the results from 266 participants, including 120 males, with an average age of 22 (21.7 ± 1.01), and 146 females with an average age of 22 (21.6 ± 1.04). There were no significant differences between males and females in age (*t* = 0.77, *P* = 0.44), IQ (*t* = −0.90, *P* = 0.37), and SES (*t* = −1.44, *P* = 0.15), as seen in Table 1.

**Table 1:** Demographic characteristics of participants.

	All *n* = 266		Males *n* = 120		Females *n* = 146		Sex differences (two-tailed *t-*test)	
	Mean	SD	Mean	SD	Mean	SD	*t*	*P*
Antisocial behavior	32.77	7.06	35.03	6.61	30.91	6.91	4.93***	1.45 *×* 10^−6^
Age	21.65	1.03	21.7	1.01	21.6	1.04	0.77	0.44
IQ	25.95	4.03	25.71	4.54	26.16	3.57	−0.90	0.37
SES	4.97	1.61	4.82	1.53	5.1	1.67	−1.44	0.15
Global GMV	0.47	0.04	0.50	0.04	0.46	0.03	9.43***	2.19 *×* 10^−18^

Note: GMV, gray matter volume. ***P < 0.001.

### Behavioral measurements

#### Antisocial behavior

The self-reported antisocial behavior was measured using the antisocial behavior subscale of the Behavioral Indicators of Conscientiousness (BIC) by Jackson *et al*. (2010). The BIC is used to measure behaviors related to conscientiousness, including impulsivity, organization, cleanliness, antisocial, and other seven subscales. The antisocial subscale contains 23 items in total, sampling antisocial behaviors in daily life, such as breaking rules in a game. The antisocial subscale has a significant negative correlation with industriousness, organization, impulsive control, and responsiveness, as well as a significant negative correlation with the agreeableness in the Big Five personality (*r* = −0.45) (Jackson *et al*., 2010). In this study, we used 15 items from it because the other eight items do not describe the the sample we tested and therefore could affect the test validity: it is rare to see Chinese students drinking during working hours and swearing when talking to the boss. As such, the internal consistency coefficient of the 15-item questionnaire remains high: Cronbach's *α* = 0.8. During the test, participants were asked to answer the frequency of their engagement in many different antisocial behaviors using a six-point scale to score from 1 “never” to 6 “always.” A higher score means more antisocial behavior, and a lower score means less antisocial behavior.

#### IQ

The intelligence test was performed with the Raven's Advance Progress Matrices (Carpenter *et al*., 1990). It is suited to college students as they have proven above-average intelligence. The test included a series of geometric graphs arranged in a logical sequence and the participants had to choose the consequent graph. There were 48 items in the test: 12 practice items and 36 test items. We took the normalized scores in the subsequent behavioral–brain correlational analysis.

#### SES

The SES was measured with the MacArthur Scale of Subjective Social Status (Adler *et al*., 2000; Goodman *et al*., 2001). The scale is presented to participants with a 10-step ladder. The highest level of the ladder represents the family with the highest standard of living in the area where the tested family is located—the highest income, the highest level of education, and the highest social status; the lowest level of the ladder represents the family with the lowest standard of living in the region. The higher the level of the family, the closer it is to the family with the highest standard of living; the lower the level of the family, the closer it is to the family with the lowest standard of living. Participants were asked to draw a cross on the corresponding level based on their families’ levels in the local areas during high school, as previous literature (Moffitt *et al*., 2001) has shown that there is a high incidence of antisocial behavior during adolescence, when teenagers are vulnerable to environmental factors such as their SES.

All the statistical analyses of the behavioral data were performed with MATLAB v.R2013a (MathWorks, Natick, MA, USA).

### MRI data collection

The MRI data were collected at the Brain Imaging Center of Beijing Normal University. The scanner was a 3 T Siemens magnetic resonance scanner (Siemens 3 T scanner, MAGENTOM Trio, a Tim system), with a 12-channel phased array head coils. The magnetic resonance structural images were acquired using a three-dimensional magnetization prepared rapid gradient echo (MP-RAGE) T1 weighted sequence (TR/TE/TI = 2530/3.39/1100 ms, flip angle = 7°, FOV = 256 × 256 mm). One scan covering the whole brain yielded 128 consecutive sagittal slices with a 1 × 1 mm planar resolution and 1.33 mm thickness.

### VBM analysis

Voxel-based morphometry (VBM) analysis (Ashburner & Friston, 2000) was performed to study the neuroanatomical basis for the sex differences of antisocial behavior. The analysis was performed in SPM8 (Statistical Parametric Mapping, Wellcome Centre for Human Neuroimaging, London, UK), using standard VBM analytic procedures for the T1 images (Good *et al*., 2001; Huang *et al*., 2022; Lu *et al*., 2023; Mechelli *et al*., 2005).

First, the image quality was checked to avoid the artifacts caused by excessive head movements and the abnormal brain anatomy. Second, the origin was manually marked at the anterior commissure for each participant for better quality control. Third, we use a unified segmentation approach (Ashburner & Friston, 2005) to segment the images into gray matter, white matter, cerebrospinal fluid, and the other parts (e.g. skull and scalp). Fourth, the gray matter images were realigned, resampled, and spatially normalized in the MNI152 space using the study-specific template generated through the "diffeomorphic anatomical registration through exponential lie algebra method" (Ashburner, 2007). Fifth, the gray matter volume value on each voxel was adjusted by multiplying the Jacobian coefficient resulting from the normalization process to retain the volume of the tissues at a relatively constant level after warping. Sixth, the generated value map was Gaussian smoothed with a full-width at half-maximum at 8 mm. Finally, as none of the reported brain regions from previous antisocial behavior studies were located in the cerebellum, we masked out the cerebellum using a gray matter probability threshold of 0.2. Thus, for each voxel in the cerebrum, one value of the gray matter volume was prepared for the subsequent regression analyses.

### Regression analyses

The regression analyses were performed in the FSL (http://www.fmrib.ox.ac.uk/fsl/index.html) (Smith *et al*., 2004).

First, to find out whether there were any neuroanatomical correlates of antisocial behavior, regardless of sex differences, we mixed male and female participants and constructed a general linear model (GLM). In the GLM model. We set the antisocial behavior as the only variable of interest; age, IQ, SES, and the averaged total cerebral gray matter volume were variables of non-interest. The regional gray matter volume was taken as the dependent variable.

More importantly, as we hypothesized that males and females have different antisocial behavior-related brain morphometry, two identical independent GLMs were made to fit the relationships between antisocial behavior and regional gray matter in males and females, respectively. The antisocial behavior was set as the variable of interest, and age, IQ, SES, and the averaged total cerebral gray matter volume were set as variables of non-interest as well.

Thus, the coefficients of the antisocial behavior in the GLMs stand for the degree to which the regional gray matter volume covaries with the antisocial behavior. As we were mainly interested in finding brain regions sensitive to the differences in males’ and females’ antisocial behavior, the antisocial coefficients for the males and females were compared using the *F*-test. For the multiple comparison correction, first, we applied a threshold of *P* < 0.01 voxel-wise for the whole brain.

We conducted an exploratory whole-brain analysis rather than functional region of interest analysis due to the inconsistent results in previous studies (Fairchild *et al*., 2013; Michalska *et al*., 2015; Raine *et al*., 2011; Smaragdi *et al*., 2017). For the cluster-level correction, first, we performed the standard Monte Carlo simulation (Ward, 2000) using the Analysis of Functional NeuroImages (http://afni.nimh.nih.gov/afni/) 3dClustsim to determine the cluster-level threshold. According to the correction, only when a cluster is bigger than the minimum cluster size determined by the Monte Carlo simulation, which was 322 voxels, could it be reported as significant, and the probability of making a type I error is <0.01. All the reported clusters appeared after the multiple comparison correction in the whole cerebrum, and the *Z* scores for each cluster were reported as well as the cluster sizes.

### *Post hoc* visualization

To see how exactly males’ and females’ gray matter volumes covary with antisocial behavior, we scatter-plotted the correlation of antisocial behavior scores and the gray matter volumes for males and females separately in the clusters acquired in the previous step. The correlational plots have also controlled for age, IQ, SES, and the averaged total cerebral gray matter volume.

## Results

### Sex differences in antisocial behavior

**Figure 1:**
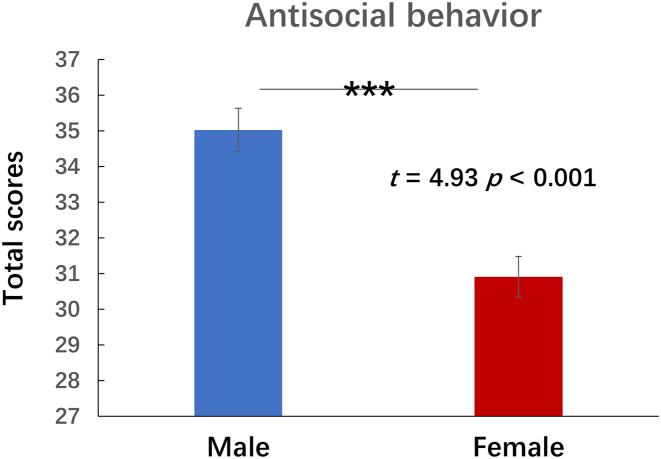
Sex differences in antisocial behavior. The *y*-axis shows the participants’ total antisocial scores from the BIC, measuring the frequency of a variety of antisocial behaviors; the two bars on the *x*-axis denote male (in blue) and female (in red) groups. *** *P* < 0.001.

Consistent with previous studies (Alegria *et al*., 2013; Cale & Lilienfeld, 2002; Moffitt *et al*., 2001; Raine *et al*., 2011), we found sex differences in antisocial behaviors (*t* = 4.93, P = 1.45 *×* 10^−6^, two-tailed test). On average, males had higher antisocial behavior scores than females (35.03 > 30.91), as shown in Fig. 1.

### Antisocial behavior

Overall, the individual differences among the total 266 participants were by no means small: the minimum was 15 and the maximum 49. The average score was 32.77, with a standard deviation of 7.06. The overall distribution follows a normal distribution, as the Kolmogorov–Smirnov test indicated [*D* (266) = 0.07, *P* = 0.14].

For the 120 male participants, the minimum score was 19, the maximum was 49, and the average was 35.03, with a standard deviation of 6.61, following a normal distribution [(*D* (120) = 0.06, *P* = 0.81].

For the 146 female participants, the minimum was 15, the maximum was 47, the average was 30.91, with a standard deviation of 6.91. It followed a normal distribution [*D* (146) = 0.10, *P* = 0.13] as well.

### Regression results

#### Sex differences in antisocial behavior

Sex and antisocial behavior have shown significant interactions on the regional gray matter volume of the brain. From Table 2, we can see that these brain regions were reported in important social cognitive and emotional functions, such as the posterior part of the temporal gyrus extending to angular gyrus, especially around the posterior superior temporal sulcus (pSTS) (peak coordinates: 58, −44,18; cluster size 4272 mm^3^), the middle temporal gyrus (MTG), which extended to the inferior temporal part (peak coordinates: 66, −10, −26; cluster size 2648 mm^3^), and the precuneus (PC) (peak coordinates: 6, −68, 36; cluster size 3296 mm^3^).

**Table 2:** Brain regions showing sex differences relating to antisocial behavior.

		Peak coordinates (MNI)				Cluster size (mm^3^)
Anatomical location	Hemisphere	*x*	*y*	*z*	*Z*	
pSTS	R	58	−44	18	4.32	4272
MTG	R	66	−10	−26	3.51	2648
Precuneus	L and R	6	−68	36	3.50	3296

Note: R: right. L: left.

**Figure 2:**
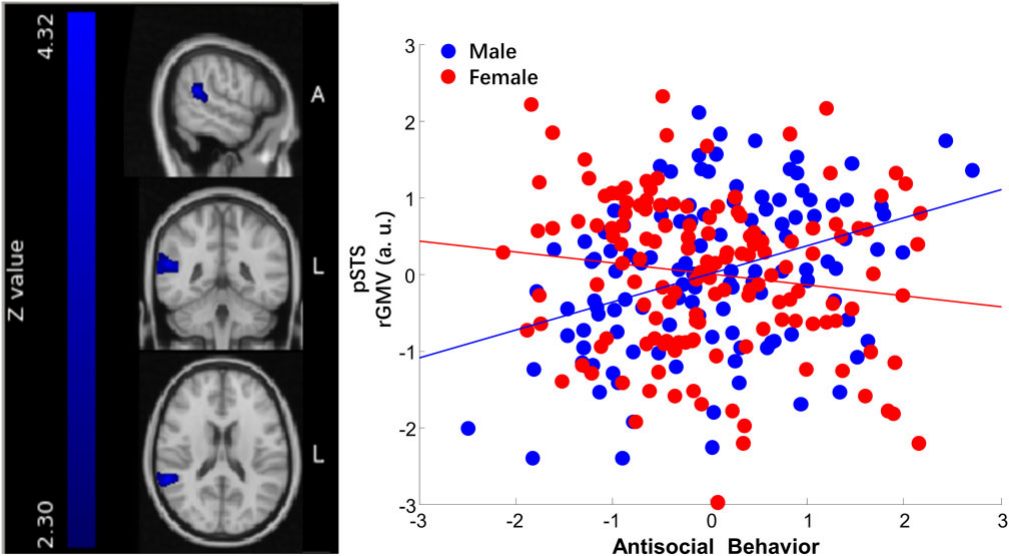
Sex and antisocial behavior interaction in pSTS. The left panel of the figure is the right pSTS cluster (peak point: 58 −44 18, cluster size 4272 mm^3^). On the right panel are the scatter plots depicting the correlations of individual differences in antisocial behavior and the average gray matter volume within the cluster in males and females. Each blue dot in the scatter plot represents each male participant given his scores on the *x*- and *y*-axes. Similarly, each red dot represents each female participant. The blue straight line stands for the correlation of antisocial behavior and the cluster gray matter volume in males, controlling for age, IQ, SES, and the total cerebral gray matter volume (*r* = 0.36, *P* = 6.50 *×* 10^−5^). The red straight line stands for the correlation of antisocial behavior and the cluster gray matter volume in females, controlling for the same factors as stated above (*r* = −0.14, *P* = 0.10).

First, the biggest interaction effect was around the pSTS. We can see a significant positive correlation (*r =* 0.36, *P* = 6.50 *×* 10^−5^) between males’ antisocial behavior and the gray matter volume in this cluster (see Fig. 2). However, this correlation in females was the opposite (*r =* −0.14, *P* = 0.10). Therefore, increased gray matter volume in the pSTS was significantly related to increased antisocial behavior in males, yet a decreased trend for antisocial behavior in females.

**Figure 3:**
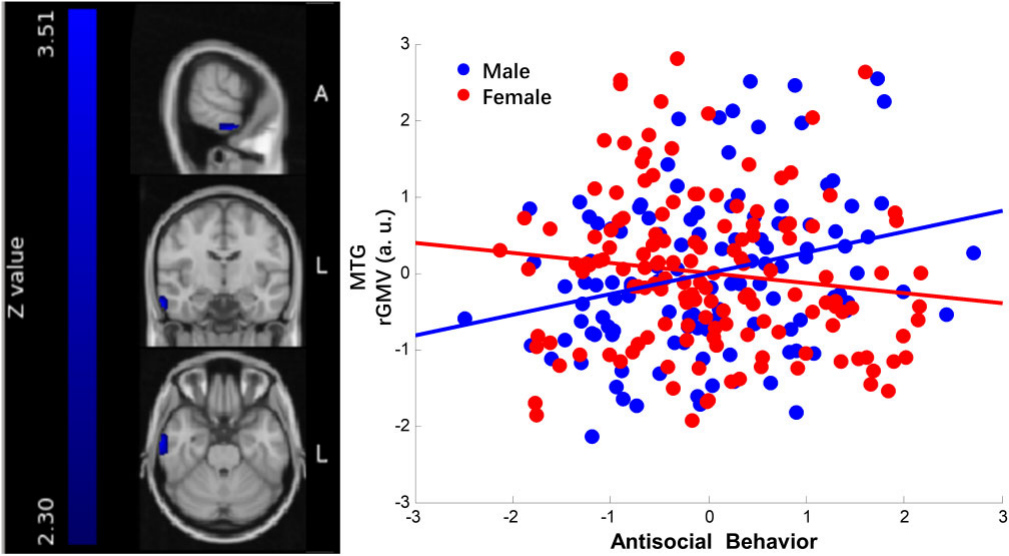
Sex and antisocial behavior interaction in MTG. The left panel of the figure is the right MTG cluster (peak point: 66 −10 −26, cluster size 2648 mm^3^). On the right panel are the scatter plots depicting the correlations of individual differences in antisocial behavior and the average gray matter volume within the cluster in males and females. Each blue dot in the scatter plot represents each male participant given his scores on the *x*- and *y*-axes. Similarly, each red dot represents each female participant. The blue straight line stands for the correlation of antisocial behavior and the cluster gray matter volume in males, controlling for age, IQ, SES, and the total cerebral gray matter volume (*r* = 0.27, *P* = 0.004). The red straight line stands for the correlation of antisocial behavior and the cluster gray matter volume in females, controlling for the same factors as stated above (*r* = −0.13, *P* = 0.12).

Similarly, in the MTG, the correlation between males’ antisocial behavior and the gray matter volume within the cluster was 0.27 (*P* = 0.004), showing that a greater gray matter volume in the MTG cluster was associated with more antisocial behavior in males. On the contrary, in females it showed a negative trend (*r* = −0.13, *P* = 0.12). The plot is shown in Fig. 3.

**Figure 4:**
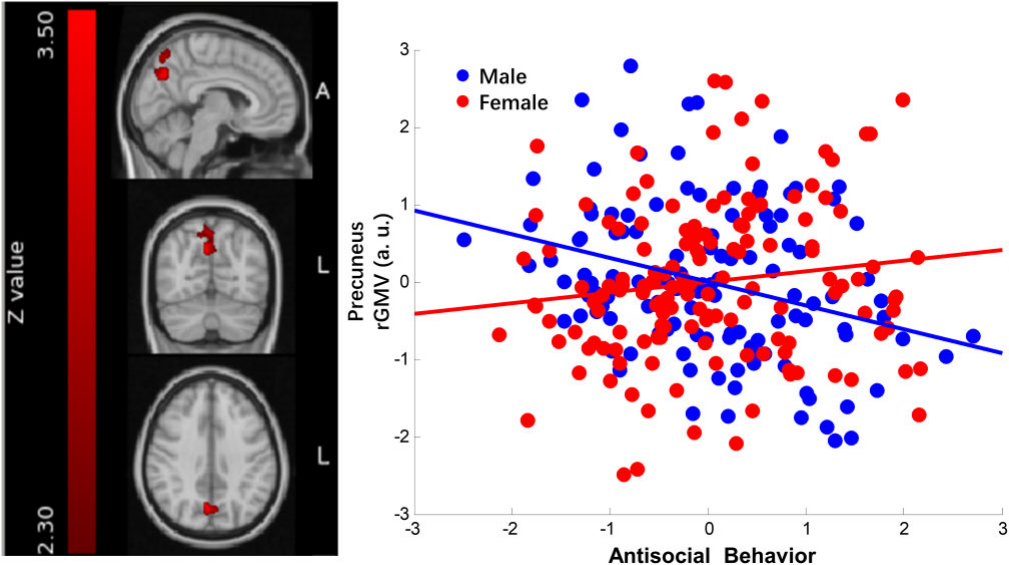
Sex and antisocial behavior interaction in precuneus. The left panel of the figure is the precuneus cluster (peak point: 6 −68 36, cluster size 3296 mm^3^). On the right panel are the scatter plots depicting the correlations of individual differences in antisocial behavior and the average gray matter volume within the cluster in males and females. Each blue dot in the scatter plot represents each male participant given his scores on the *x*- and *y*-axes. Similarly, each red dot represents each female participant. The blue straight line stands for the correlation of antisocial behavior and the cluster gray matter volume in males, controlling for age, IQ, SES, and the total cerebral gray matter volume (*r* = −0.31; *P* = 7.89 *×* 10^−04^). The red straight line stands for the correlation of antisocial behavior and the cluster gray matter volume in females, controlling for the same factors as stated above (*r* = 0.14, *P* = 0.11).

However, in the PC, the male antisocial behavior and regional gray matter volume showed a significant negative correlation (*r* = −0.31; *P* = 7.89 *×* 10^−04^); whereas in females, the correlation was positive nevertheless not significant (*r* = 0.14, *P* = 0.11). As shown in Fig. 4, such results showed that in the precuneus, more antisocial behavior was associated with reduced gray matter volume in males; yet for females more antisocial behavior was associated with increased gray matter volume as a trend.

#### Antisocial behavior

**Figure 5:**
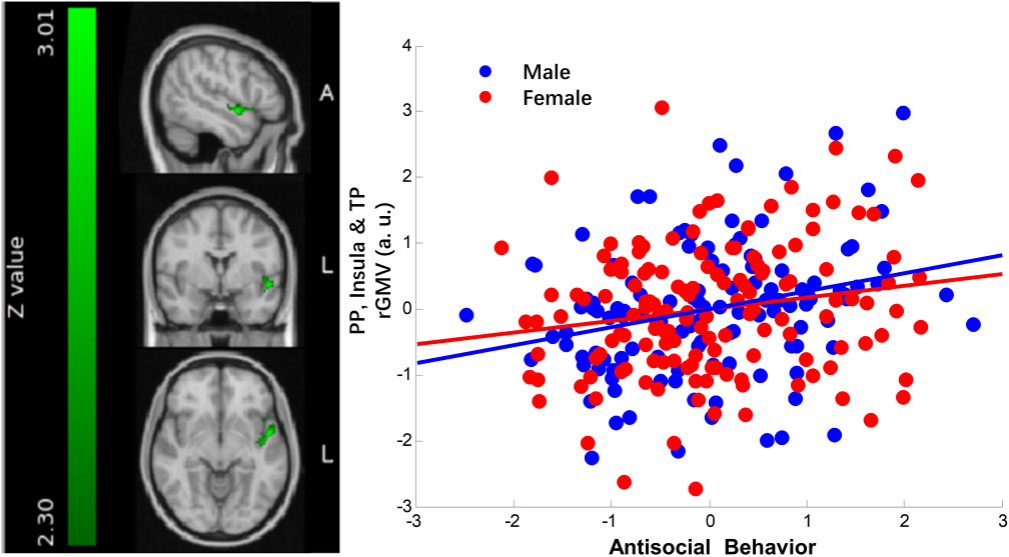
The main effect of antisocial behavior. The left panel of the figure shows the cluster in the planum polare, extending to the insula and temporal pole, which is depicted in green (cluster size = 2768 mm^3^). The center of the cluster is at the peak point (MNI coordinates −50 0 −8). The scatter plots on the right panel depict the correlations of individual differences in antisocial behavior and the average gray matter volume within the cluster in males and females. Each blue dot in the scatter plot represents each male participant given his scores on the *x*- and *y*-axes. Similarly, each red dot represents each female participant. The blue straight line stands for the correlation of antisocial behavior and the cluster gray matter volume in males, controlling for age, IQ, SES, and the total cerebral gray matter volume (*r* = 0.27, *P* = 0.0029). The red straight line stands for the correlation of antisocial behavior and the cluster gray matter volume in females, controlling for the same factors as stated above (*r* = 0.18, *P* = 0.03).

In line with previous studies (Aoki *et al*., 2013; De Brito *et al*., 2009; Raine, 2019), we found that antisocial behavior was related to the gray matter volume in the anterior part of the temporal lobe and insula. Specifically, the cluster size is 2768 mm^3^ (peak: −50 0 −8) as described in Table 3. On the temporal lobe, the cluster extends from the planum polare, the anterior part of the superior temporal gyrus, to the temporal pole; meanwhile, the cluster also extends to the insula from its posterior to the anterior part. The cluster centered at its peak location is illustrated in Fig. 5. The results showed that there is a common neuroanatomical basis for antisocial behavior in both males and females in a large non-clinical sample of young adults, and this basis is mainly located in the left anterior part of the superior temporal gyrus and the insula.

**Table 3:** Antisocial behavior correlated brain structures.

		Peak coordinates (MNI)				*Post hoc* (*r*)		Cluster size (mm^3^)
Anatomical location	Hemisphere	*x*	*y*	*z*	*Z*	Males	Females	
Planum polare, insula, and temporal pole	L	−50	0	−8	3.01	0.27**	0.18*	2768

Note: L: left. **P < 0.01 (*P* = 0.0029); *P < 0.05 (*P* = 0.0337).

*Post hoc* analyses showed that, for males, after controlling for age, IQ, SES, and the total cerebral gray matter volume, there was a significant correlation (*r* = 0.27, *P* = 0.0029) of antisocial behavior and the gray matter volume of the cluster. Similarly, for females such a correlation was also significant (*r* = 0.18, *P* = 0.0337). This indicated that both male and female antisocial behavior could be modulated by this region.

## Discussion

The current study provides the first evidence of sex differences in brain morphometry related to self-reported antisocial behavior in a large non-clinical sample of young adults. First, we have replicated the sex differences in antisocial behavior showing that males demonstrated more antisocial behavior than females. Moreover, further results revealed that the pSTS, MTG, and PC were the primary brain regions where males and females exhibited different patterns of correlations between antisocial behavior and regional gray matter volume. Additionally, we have also found the common neural substrates of antisocial behavior for both males and females around the insula. As these regions related to sex differences in antisocial behavior are generally involved in several crucial social functions, such as theory of mind, empathy, and moral judgment, it indicates that the sex differences in these functions may influence antisocial behavior by shaping the brain's gray matter volume during development.

The sex differences in antisocial behavior have been well-established and many studies have tried to explain the phenomenon by exploring the risk factors behind it. Basically, there are three main explanations for why males are more antisocial than females (Moffitt *et al*., 2001). First, males are more susceptible to risk factors than females, as reflected in the higher correlation between antisocial behavior and risk factors in males than in females. This is a similar to our findings the correlations between regional gray matter volume and antisocial behavior are always stronger in males than in females. The second explanation is that males are exposed to higher levels of risk factors than females: for childhood problematic behaviors, males have higher prevalence of ADHD than females; in peer relationships, males have more misbehaving peers. Finally, the differences in personalities and propensities between males and females may also be the cause. In personality traits closely related to antisocial behavior, there are significant sex differences: e.g., males have more negative emotions and are worse at self-control. The differences between males and females in these two aspects can explain 96% of the differences in the antisocial behavior in adolescents, and 78% of the differences in the chance of developing a conduct disorder.

For the common neural basis, we found that the brain region generally associated with antisocial behavior was around the insula and temporal pole. The findings on the insula were consistent with a former VBM study in males but not females (Fairchild *et al*., 2013): males with conduct disorder had more gray matter volume in the left anterior insula than normal controls while females had less. The different result in females could be due to small sample size and high comorbidity of ADHD and major depressive disorder (MDD) in adolescent girls with conduct disorder, according to the authors. Therefore, our results from a large non-clinical sample might be more likely to provide a stable normative effect. The anterior part of the insula is a classic functional area in emotional processing. It is responsible for interoceptive feelings and experiences participating in various emotional processing such as anger and fear (Craig, 2009). It is also strongly connected with the amygdala (Meyer-Lindenberg & Tost, 2012; Naqvi & Bechara, 2009; Sescousse *et al*., 2013). Antisocial offenders have deficits in emotional processing: in fMRI studies, antisocial offenders have less active brain activities in regions involved in emotional processing than normal participants, and are less sensitive to the negative emotions of others (Herpertz *et al*., 2008; Sadeh *et al*., 2011). This neurobiological basis indicates that they are more likely to ignore others’ emotions, hurt others, or violate socially accepted moral standards. Furthermore, the anterior insula and temporal pole are also the core brain regions of empathy (de Vignemont & Singer, 2006; Fan *et al*., 2011; Morita *et al*., 2013; Ponz *et al*., 2013; Vogt, 2005). Empathy is an important prosocial quality. It includes the cognitive ability to see the other's perspective, and also the emotional ability to share another's emotional experience. The deficit in empathy is a common cause for many mental illnesses and social maladaptation. As antisocial behavior is characterized as callous and unemotional, showing a lack of concern for others' suffering, which is closely associated with the functions of the anterior insula and temporal pole, it could be that the antisocial behavior is the manifestation of the dysfunction in these core brain regions. Taken together, the functional deficits in emotional processing and empathy may affect antisocial behavior in general.

More importantly, we found sex dimorphic cortical brain volumes associated with antisocial behavior around the pSTS, MTG, and precuneus.

First, the right pSTS is located at the posterior part of the superior temporal sulcus and is connected to the temporoparietal junction (TPJ) and the inferior parietal lobe. It is frequently reported in various social cognitive brain imaging studies. Fundamentally, the pSTS is part of the human mirror neuron system and responsible for understanding facial expressions, actions, and the recognition of action intentions (Rizzolatti & Craighero, 2004; Van Overwalle & Baetens, 2009). Based on this, it is also involved in higher-level social cognitive processing such as social perception, theory of mind, empathy, moral judgment, and decision-making. Our results showed that there was a significant positive correlation between male antisocial behavior and the gray matter volume around the pSTS. This is consistent with previous studies reporting increased gray matter volume in boys with conduct disorder and callous–unemotional traits (De Brito *et al*., 2009). Additionally, Ibrahim *et al*. (2021) and Smaragdi *et al*. (2017) also reported a close-by brain region, the supramarginal gyrus, which exhibited a sex-by-diagnosis interaction on cortical thickness in children and adolescents diagnosed with disruptive or conduct disorders. Nevertheless, this interaction manifested as a reduction in cortical thickness in males, which was contrary to our findings. The inconsistencies could be due to the comorbidity mentioned in the articles, such as ADHD, depression, and also the differences in substance use, medication, and age of onset of the diseases. Despite the differences, our study and these previous studies in patients commonly found the crucial location, which is around the pSTS. Moreover, this region was reported in the reviews of the neural basis of antisocial behavior in 2008 and 2013 (Aoki *et al*., 2013; Raine, 2008). According to the reviews, male antisocial behavior may be regulated by the pSTS through several social cognitive abilities such as social perception, theory of mind, empathy, and moral judgment. Deficits in these functions could lead to antisocial behavior. For example, poor social perception and theory of mind abilities may cause wrong attribution of others' intentions, resulting in aggressive behavior. Lack of empathy may lead to ignorance of others’ sufferings and thus unable to stop hurting behavior. It also happened that males were less empathetic than females (Adenzato *et al*., 2017), which could be an explanation of the sex differences in brain structures related to empathy. The finding supported the neuromoral theory of antisocial behavior (Raine, 2019). Moreover, our findings refined previous reviewsby showing that this correlation was more specific to males, while females showed the opposite trend and were less affected. One possible argument could be that, since females showed less antisocial behavior than males, the non-significant correlation could be due to the lack of individual differences in the distribution of antisocial behavior in females. However, from the scatter plot in Fig.   2, we can see that the individual differences in female's antisocial behavior were no less than in males: all the female participants shown in red dots are not densely concentrated in one area. Besides, the standard deviations in males and females are also comparable. Therefore, the sex differences in pSTS gray matter volume are likely to reflect sex-selective social deficits such as empathy in males.

Moreover, our results showed that males exhibiting more antisocial behavior have more gray matter volume in the MTG. This is consistent with the previous study (Yang *et al*., 2015) that adolescents with higher psychopathic traits have higher cortical thickness in MTG, and this is specifically found in males. Besides, functional neuroimaging studies also reported abnormal neural activities in the MTG in male violent offenders (Gregory *et al*., 2015; Kumari *et al*., 2009). Similar to the pSTS, the MTG has also been frequently reported in many studies relevant to social cognitive functions. In particular, a previous meta-analysis (Bzdok *et al*., 2012) has revealed that the right MTG is one of a few robust regions commonly involved in moral cognition, theory of mind, and empathy—the other regions are the dorsomedial prefrontal cortex and bilateral TPJ. As has been discussed before, moral reasoning, theory of mind, and empathy are three crucial abilities closely related to antisocial behavior. The sex differences in these functions may result in the divergence in the development of the MTG morphometry.

Notably, as the pSTS and MTG are both within the temporal lobe, we have to acknowledge that sex differences in the development of the structures of the temporal lobe could influence antisocial behavior (Michalska *et al*., 2015). In line with the positive correlation of antisocial behavior and gray matter volume in the temporal lobe (pSTS and MTG) in males, we also found similar results in other populations: children with callous–unemotional traits and conduct disorder have more gray matter in the temporal lobe than normal children (De Brito *et al*., 2009). Children and adults may have completely different patterns of correlation due to developmental delay or functional deficits, showing increased or reduced gray matter volume in various locations in the temporal lobe. This could explain why our results in young adults differ from some of those in children (Michalska *et al*., 2015) and adolescents (Fairchild *et al*., 2013; Smaragdi *et al*., 2017).

The precuneus has been less discussed in previous neuroimaging studies of antisocial behavior, although it is an important part of the default mode network (DMN). Our study found that males showed significant gray matter volume reduction in the precuneus with the increase of antisocial behavior, which is consistent with former studies on antisocial clinical samples reporting significant loss in the gray matter volume in the precuneus (Raine *et al*., 2000). The precuneus and the posterior cingulate gyrus are adjacent to each other and form one of the core nodes of the DMN. They also have strong functional connectivity with the medial prefrontal cortex and the lateral temporal cortex (Buckner *et al*., 2008). The DMN is a set of brain regions that show spontaneous neural activities without performing any external tasks. It includes the medial prefrontal cortex, precuneus and posterior cingulate gyrus, IPL, lateral temporal cortex, and parahippocampal gyrus. These brain regions are well connected and are mainly involved in functions involving self-referential processing, such as autobiographical memory and moral decision-making (Buckner *et al*., 2008). A recent study of sex differences in adult human brains (based on > 5000 individuals) showed that males have weaker connectivity within the DMN nodes than females (Ritchie *et al*., 2018), which indirectly supports our finding in a male deficit perspective.

While the study may shed light on sex differences in brain morphometry related to self-reported antisocial behavior, several limitations warrant consideration. First, the cross-sectional design restricts the ability to establish causal relationships between brain morphometry and antisocial behavior. The lack of longitudinal data impedes the identification of developmental trajectories and precludes definitive conclusions about whether changes in brain structure precede or follow the onset of antisocial behavior. Additionally, the reliance on self-reported measures for antisocial behavior may introduce biases or underreporting, as individuals might be hesitant to disclose sensitive or socially undesirable behaviors accurately. Furthermore, the generalizability of the findings might be limited since the study predominantly focuses on young adults from a non-clinical population, which does not fully represent individuals with clinically significant antisocial behaviors. Future research employing longitudinal designs, objective measures of antisocial behaviors, and a more diverse sample could provide a more comprehensive understanding of the nuanced relationship between brain morphometry and antisocial behavior across different populations and developmental stages.

Taken together, our study identified the neuroanatomical correlates of the sex differences in antisocial behavior in a large non-clinical sample, and the effect is independent of age, IQ, SES, and total cerebral gray matter volume. As these brain regions are mainly involved in emotional processing, theory of mind, empathy, moral judgment, and self-referential processing, the sex differences in these functions might modulate antisocial behavior through the development of the brain morphometry. This study provides a neuroanatomical perspective on the male preponderance of antisocial behavior and contributes evidence to the biological basis of sex differences in antisocial behavior from a large non-clinical young adult sample.

## References

[bib1] Adenzato M, Brambilla M, Manenti R et al. (2017) Gender differences in cognitive theory of mind revealed by transcranial direct current stimulation on medial prefrontal cortex. Sci Rep. 7:41219. 28117378 10.1038/srep41219 PMC5259730

[bib2] Adler NE, Epel ES, Castellazzo G et al. (2000) Relationship of subjective and objective social status with psychological and physiological functioning: preliminary data in healthy, White women. Health Psychol. 19:586–92. 11129362 10.1037//0278-6133.19.6.586

[bib3] Alegria AA, Blanco C, Petry NM et al. (2013) Sex differences in antisocial personality disorder: results from the National Epidemiological Survey on Alcohol and Related Conditions. Personal Disord Theory Res Treat. 4:214–22. 10.1037/a0031681 PMC3767421 23544428

[bib4] Aoki Y, Inokuchi R, Nakao T et al. (2013) Neural bases of antisocial behavior: a voxel-based meta-analysis. Soc Cogn Affect Neurosci. 9:1223–31. 23926170 10.1093/scan/nst104 PMC4127028

[bib5] Ashburner J (2007) A fast diffeomorphic image registration algorithm. Neuroimage. 38:95–113. 17761438 10.1016/j.neuroimage.2007.07.007

[bib6] Ashburner J, Friston KJ (2000) Voxel-based morphometry—the methods. Neuroimage. 11:805–21. 10860804 10.1006/nimg.2000.0582

[bib7] Ashburner J, Friston KJ (2005) Unified segmentation. Neuroimage. 26:839–51. 15955494 10.1016/j.neuroimage.2005.02.018

[bib8] Bennett S, Farrington DP, Huesmann LR (2005) Explaining gender differences in crime and violence: the importance of social cognitive skills. Aggress Violent Behav. 10:263–88.

[bib9] Blair RJR (2001) Neurocognitive models of aggression, the antisocial personality disorders, and psychopathy. J Neurol Neurosurg Psychiatry. 71:727–31. 11723191 10.1136/jnnp.71.6.727 PMC1737625

[bib10] Buckner RL, Andrews‐Hanna JR, Schacter DL (2008) The brain's default network. Ann NY Acad Sci. 1124:1–38. 18400922 10.1196/annals.1440.011

[bib11] Burt SA, Slawinski BL, Carsten EE et al. (2019) How should we understand the absence of sex differences in the genetic and environmental origins of antisocial behavior?. Psychol Med. 49:1600. 30957728 10.1017/S0033291719000771 PMC7232938

[bib12] Bzdok D, Schilbach L, Vogeley K et al. (2012) Parsing the neural correlates of moral cognition: ALE meta-analysis on morality, theory of mind, and empathy. Brain Struct Funct. 217:783–96. 22270812 10.1007/s00429-012-0380-y PMC3445793

[bib13] Cale EM, Lilienfeld SO (2002) Sex differences in psychopathy and antisocial personality disorder. Clin Psychol Rev. 22:1179–207. 12436810 10.1016/s0272-7358(01)00125-8

[bib14] Carpenter PA, Just MA, Shell P (1990) What one intelligence test measures: a theoretical account of the processing in the Raven Progressive Matrices Test. Psychol Rev. 97:404–31. 2381998

[bib15] (Bud) Craig AD (2009) How do you feel—now? The anterior insula and human awareness. Nat Rev Neurosci. 10:59–70. 19096369 10.1038/nrn2555

[bib16] Dabbs JM, Morris R (1990) Testosterone, social class, and antisocial behavior in a sample of 4,462 men. Psychol Sci. 1:209–11.

[bib17] De Brito SA, Mechelli A, Wilke M et al. (2009) Size matters: increased grey matter in boys with conduct problems and callous–unemotional traits. Brain. 132:843–52. 19293245 10.1093/brain/awp011

[bib18] De Vignemont F, Singer T (2006) The empathic brain: how, when and why?. Trends Cogn Sci. 10:435–41. 16949331 10.1016/j.tics.2006.08.008

[bib19] Fairchild G, Hagan CC, Walsh ND et al. (2013) Brain structure abnormalities in adolescent girls with conduct disorder. Child Psychol Psychiatry. 54:86–95. 10.1111/j.1469-7610.2012.02617.x PMC3562487 23082797

[bib20] Fan Y, Duncan NW, de Greck M et al. (2011) Is there a core neural network in empathy? An fMRI based quantitative meta-analysis. Neurosci Biobehav Rev. 35:903–11. 20974173 10.1016/j.neubiorev.2010.10.009

[bib21] Good CD, Johnsrude I, Ashburner J et al. (2001) Cerebral asymmetry and the effects of sex and handedness on brain structure: a voxel-based morphometric analysis of 465 normal adult human brains. Neuroimage. 14:685–700. 11506541 10.1006/nimg.2001.0857

[bib22] Goodman E, Adler NE, Kawachi I et al. (2001) Adolescents' perceptions of social status: development and evaluation of a new indicator. Pediatrics. 108:e31. 11483841 10.1542/peds.108.2.e31

[bib23] Gregory S, Blair RJ, Ffytche D et al. (2015) Punishment and psychopathy: a case-control functional MRI investigation of reinforcement learning in violent antisocial personality disordered men. Lancet Psychiatry. 2:153–60. 26359751 10.1016/S2215-0366(14)00071-6

[bib24] Herpertz SC, Huebner T, Marx I et al. (2008) Emotional processing in male adolescents with childhood-onset conduct disorder. Child Psychol Psychiatry. 49:781–91. 10.1111/j.1469-7610.2008.01905.x 18598245

[bib25] Huang H, Zheng S, Yang Z et al. (2022) Voxel-based morphometry and a deep learning model for the diagnosis of early Alzheimer's disease based on cerebral gray matter changes. Cereb Cortex. 33:754–63. 10.1093/cercor/bhac099 PMC9890469 35301516

[bib26] Ibrahim K, Kalvin C, Li F et al. (2021) Sex differences in medial prefrontal and parietal cortex structure in children with disruptive behavior. Dev Cogn Neurosci. 47:100884. 33254067 10.1016/j.dcn.2020.100884 PMC7704291

[bib27] Jackson JJ, Wood D, Bogg T et al. (2010) What do conscientious people do? Development and validation of the Behavioral indicators of conscientiousness (BIC). J Res Personal. 44:501–11. 10.1016/j.jrp.2010.06.005 PMC3028204 21278818

[bib28] Kumari V, Das M, Taylor PJ et al. (2009) Neural and behavioural responses to threat in men with a history of serious violence and schizophrenia or antisocial personality disorder. Schizophr Res. 110:47–58. 19230621 10.1016/j.schres.2009.01.009

[bib29] Lu F, Cui Q, Chen Y et al. (2023) Insular-associated causal network of structural covariance evaluating progressive gray matter changes in major depressive disorder. Cereb Cortex. 33:831–43. 35357431 10.1093/cercor/bhac105

[bib30] Mechelli A, Price C, Friston K et al. (2005) Voxel-based morphometry of the human brain: methods and applications. CMIR. 1:105–13.

[bib31] Meyer-Lindenberg A, Tost H (2012) Neural mechanisms of social risk for psychiatric disorders. Nat Neurosci. 15:663–8. 22504349 10.1038/nn.3083

[bib32] Michalska KJ, Decety J, Zeffiro TA et al. (2015) Association of regional gray matter volumes in the brain with disruptive behavior disorders in male and female children. NeuroImage: Clinical. 7:252–7. 25610787 10.1016/j.nicl.2014.12.012 PMC4300012

[bib33] Moffitt TE, Caspi A, Rutter M et al. (2001) Sex Differences in Antisocial Behaviour: Conduct Disorder, Delinquency, and Violence in the Dunedin Longitudinal Study. Cambridge, United Kingdom: Cambridge University Press.

[bib34] Morita T, Tanabe HC, Sasaki AT et al. (2013) The anterior insular and anterior cingulate cortices in emotional processing for self-face recognition. Soc Cogn Affect Neurosci. 9:570–9. 23377900 10.1093/scan/nst011 PMC4014092

[bib35] Naqvi NH, Bechara A (2009) The hidden island of addiction: the insula. Trends Neurosci. 32:56–67. 18986715 10.1016/j.tins.2008.09.009 PMC3698860

[bib36] Patterson GR, Debaryshe BD, Ramsey E (1989) A developmental perspective on antisocial behavior. Am Psychol. 44:329–35. 2653143 10.1037//0003-066x.44.2.329

[bib37] Ponz A, Montant M, Liegeois-Chauvel C et al. (2013) Emotion processing in words: a test of the neural re-use hypothesis using surface and intracranial EEG. Soc Cogn Affect Neurosci. 9:619–27. 23482627 10.1093/scan/nst034 PMC4014107

[bib38] Raine A, Lencz T, Bihrle S et al. (2000) Reduced prefrontal gray matter volume and reduced autonomic activity in antisocial personality disorder. Arch Gen Psychiatry. 57:119. 10665614 10.1001/archpsyc.57.2.119

[bib39] Raine A, Yang Y, Narr KL et al. (2011) Sex differences in orbitofrontal gray as a partial explanation for sex differences in antisocial personality. Mol Psychiatry. 16:227–36. 20029391 10.1038/mp.2009.136 PMC3008752

[bib40] Raine A (2008) From genes to brain to antisocial behavior. Curr Dir Psychol Sci. 17:323–8.

[bib41] Raine A (2019) The neuromoral theory of antisocial, violent, and psychopathic behavior. Psychiatry Research. 277:64–69. 30473129 10.1016/j.psychres.2018.11.025

[bib42] Ritchie SJ, Cox SR, Shen X et al. (2018) Sex differences in the adult human brain: evidence from 5216 UK Biobank participants. Cereb Cortex. 28:2959–75. 29771288 10.1093/cercor/bhy109 PMC6041980

[bib43] Rizzolatti G, Craighero L (2004) The mirror-neuron system. Annu Rev Neurosci. 27:169–92. 15217330 10.1146/annurev.neuro.27.070203.144230

[bib44] Sadeh N, Spielberg JM, Heller W et al. (2011) Emotion disrupts neural activity during selective attention in psychopathy. Soc Cognit Affect Neurosci. 8:235–46. 22210673 10.1093/scan/nsr092 PMC3594718

[bib45] Sescousse G, Caldú X, Segura B et al. (2013) Processing of primary and secondary rewards: a quantitative meta-analysis and review of human functional neuroimaging studies. Neurosci Biobehav Rev. 37:681–96. 23415703 10.1016/j.neubiorev.2013.02.002

[bib46] Smaragdi A, Cornwell H, Toschi N et al. (2017) Sex differences in the relationship between conduct disorder and cortical structure in adolescents. J Am Acad Child Adolesc Psychiatry. 56:703–12. 28735700 10.1016/j.jaac.2017.05.015

[bib47] Smith SM, Jenkinson M, Woolrich MW et al. (2004) Advances in functional and structural MR image analysis and implementation as FSL. Neuroimage. 23:S208–19. 15501092 10.1016/j.neuroimage.2004.07.051

[bib48] Van Overwalle F, Baetens K (2009) Understanding others' actions and goals by mirror and mentalizing systems: a meta-analysis. Neuroimage. 48:564–84. 19524046 10.1016/j.neuroimage.2009.06.009

[bib49] Vogt. (2005) Pain and emotion interactions in subregions of the cingulate gyrus. Nat Rev Neurosci. 6:533–44. 15995724 10.1038/nrn1704 PMC2659949

[bib50] Ward BD (2000) Simultaneous inference for fMRI data. AFNI 3d Deconvolve Documentation. Wisconsin: Medical College of Wisconsin. https://www.frontiersin.org/articles/10.3389/fnbeh.2015.00305/full#B91.

[bib51] Yang Y, Wang P, Baker LA et al. (2015) Thicker temporal cortex associates with a developmental trajectory for psychopathic traits in adolescents. PLoS ONE. 10:e0127025. 26017779 10.1371/journal.pone.0127025 PMC4446360

[bib52] Yildirim BO, Derksen JJL (2012). A review on the relationship between testosterone and life-course persistent antisocial behavior. Psychiatry Res. 200:984–1010. 22925371 10.1016/j.psychres.2012.07.044

